# Urinary potassium is a potential biomarker of disease activity in Ulcerative colitis and displays in vitro immunotolerant role

**DOI:** 10.1038/s41598-017-18046-x

**Published:** 2017-12-22

**Authors:** Sandeep Goyal, Ritika Rampal, Saurabh Kedia, Sandeep Mahajan, Sawan Bopanna, Devesh P. Yadav, Saransh Jain, Amit Kumar Singh, Md. Nahidul Wari, Govind Makharia, Amit Awasthi, Vineet Ahuja

**Affiliations:** 1 Department of Gastroenterology and Human Nutrition, All India Institute of Medical Sciences, New Delhi, 110029 India; 2 Department of Nephrology, All India Institute of Medical Sciences, New Delhi, 110029 India; 3 Center for Human Microbial Ecology, Translation Health Science and Technology Institute, Faridabad, Haryana 121001 India

## Abstract

We evaluated the *in-vitro* effect of potassium on CD4^+^ T cells and the role of urinary potassium as a potential biomarker of disease activity in patients with ulcerative colitis (UC). This prospective observational cohort study included healthy controls (n = 18) and UC patients [n = 30, median age: 40 (IQR: 28–46) years, 17 males)] with active disease(assessed by Mayo score) from September 2015–May 2016. Twenty-four hours urinary potassium along with fecal calprotectin (FCP) were estimated in UC patients (at baseline and follow-up after 3–6 months) and controls. In healthy volunteers, we also assessed the effect of potassium on CD4^+^ T cells differentiated in the presence of Th17 polarizing condition. UC patients had significantly higher FCP (368.2 ± 443.04 vs 12.44 ± 27.51, p < 0.001) and significantly lower urinary potassium (26.6 ± 16.9 vs 46.89 ± 35.91, p = 0.01) levels than controls. At follow-up, a significant increase in urinary potassium among patients who had clinical response [n = 22, 21.4 (14.4–39.7) to 36.5 (20.5–61.6), p = 0.04] and remission [n = 12, 18.7 (9.1–34.3) to 36.5 (23.4–70.5), p = 0.05] was accompanied with a parallel decline in FCP. On *in-vitro* analysis, potassium under Th17 polarizing conditions significantly inhibited IL-17 and interferon-$\gamma$ expression while favoring the induction of FoxP3^+^ T cells. Therefore, urinary potassium levels are inversely associated with disease activity in UC with *in-vitro* data supporting an immune-tolerant role of potassium.

## Introduction

The disease course of ulcerative colitis (UC) is characterized by concurrent periods of relapses and remissions and these relapses often occur in an unpredictable manner^1^. The goals of IBD therapy have not just expanded from clinical remission to mucosal healing but also improvement in quality of life, reduction in surgeries and hospitalizations, and regular assessment of disease activity are considered important to accomplish this goal. The assessment of disease activity at index presentation is usually done by endoscopic examination. However, assessment at follow up becomes difficult as repetitive semi invasive and costly investigations such as endoscopy are unacceptable especially to the patients.

In the past decades, various laboratory markers based on inflammation and acute phase response have been studied as objective parameters to assess disease activity and avoid invasive endoscopic procedures^2,3^. Biomarkers which have been used can be divided into serological and fecal categories. Of the serological markers, C-reactive protein (CRP) is the most widely studied parameter, but is limited by being a non-specific marker of inflammation^4,5^. Potentially, fecal markers have the advantage of possessing higher specificity for gastrointestinal diseases and include labeled leucocyte scintigraphy^6^, fecal calprotectin^7^, lactoferrin and neoptrin. However, their use in clinical practice is limited by the cost especially in a resource intensive setting like that in developing countries.

Apart from the genetic factors, environment plays a major role in regulating the balance between the immune tolerance and inflammation in the gut. One such factor is salt, which due to its higher consumption in the western diet, has recently emerged to play a game changing role in the gut immune response. High sodium intake and excretion (a reflection of sodium intake) has been found to be associated with increased inflammation, higher cardiovascular risks, cancer and renal inflammation in chronic kidney disease patients^8,9^. Recent murine studies have also shown that excess salt (sodium chloride) via a p38/MAPK-NFAT5-SGK1 signaling induces a highly pathogenic and stable Th17 phenotype^10^. High NaCl also inhibits IL-10 secretion^11^ and suppressive function of Foxp3+ regulatory T cells thereby promoting the proinflammatory response in the gut^12^. Moreover, high salt exacerbates the immune response by activating the pro-inflammatory M1 macrophages^13^ while alternatively reducing the activation of anti-inflammatory M2 macrophage^14^. While there is considerable expansion of knowledge for sodium dependent immune responses, the literature on the association of potassium with inflammatory responses is limited. A recent study found an inverse relationship between dietary potassium intake and risk of Crohn’s disease and UC. This suggests that potassium may be associated with attenuated gut inflammatory responses.

Therefore, the goal of this study was to elucidate the role of potassium as an anti-inflammatory marker in patients with ulcerative colitis. We investigated alterations in urinary potassium levels in patients with UC having active disease and subsequently on follow up after achieving clinical response. Finally, to support our clinical findings, we explored the *in vitro* effect of potassium on effector CD4^+^ T cells in the presence of Th17 inducing inflammatory environment.

## Materials and Methods

### Study design

A prospective observational cohort study was conducted at the inflammatory bowel disease (IBD) clinic, All India Institute of Medical Sciences (AIIMS) from September 2015 to May 2016. Eighteen healthy volunteers and thirty Ulcerative colitis patients with mild to severe disease activity as assessed by Mayo score were included in this study. Written, informed consent was taken from patient and control populations, before taking urine, fecal and blood samples. Patients with age <18 years or >75 years, pregnant women, patients with history of diabetes mellitus, chronic kidney disease, hypertension, coronary artery disease, patients on drugs which might influence the urinary potassium viz. diuretics, angiotensin converting enzyme (ACE) inhibitors, angiotensin receptor blockers (ARBs) were excluded from the study. The study protocol was approved by institutional ethics committee (IESC/T-215/05.05.15) of AIIMS. All experiments were performed in accordance with relevant guidelines and regulations.

#### Clinical information and sample collection

Clinical information was collected incorporating all baseline characteristics as well as treatment details. Data was collected for patient demographics, disease duration, type of disease and severity, treatment given and outcome. Montreal classification^15^ was used to determine the disease extent while the disease activity in UC patients were characterized using Mayo score^16^.

Serum samples from both UC patient and controls were collected for determining C-reactive protein while 24 hours urinary sample was collected for estimating urinary potassium excretion. On the same day, fecal samples were collected for measuring fecal calprotectin (FC) levels. CRP, fecal calprotectin and 24 hour urinary potassium levels were repeated in followed up UC patients after 3–6 months of treatment.

### Diagnosis of UC

Diagnosis of Ulcerative colitis was made based on the European Crohn’s and Colitis Organization (ECCO) guidelines, employing a combination of clinical, endoscopic and histological features^17^.

#### Clinical response

Defined as decline in Mayo score by ≥3 at follow up

#### Clinical remission

Mayo score <3

#### Mucosal healing

Mayo endoscopy subscore of 1 or less, with a reduction of at least 1 point from baseline

### Measurement of Fecal calprotectin levels

Stool samples were collected and frozen at −20 °C. Fecal Calprotectin levels were measured by ELISA based methods using KAPEPKT849 kit (DIAsource ImmunoAssays S.A. – Belgium). Normal cut off value was taken <47 μg/g of stool.

### Measurement of Urine electrolyte levels

5 ml of 24-hour urine samples were collected and stored at 4–8 °C until further analysis. The analysis was done by electrolyte analyzer (XI- 921; Caretium medical instrument co. ltd, Shenzen China).

## *In vitro* study: Effect of potassium on the expression profile of Th17 cells

### T cell isolation and stimulation

Peripheral venous blood was obtained from healthy volunteers in compliance with the AIIMS Institutional ethics committee protocols. Peripheral blood mononuclear cells (PBMCs) were separated by Ficoll-Paque plus (GE Healthcare, Piscataway, NJ) gradient centrifugation. The PBMCs were stained with CD4- Allophycocyanin (APC), CD25-phycoerythrin-Cy7 (PE/Cy7) and CD45 RA- Phycoerithrin (PE) (Biolegend, San Diego, USA). Naive T cells (CD4^+^ CD25^−^ CD45RA^+^) were sorted; by high speed flow cytometry with FACS AriaIII (BD Biosciences, San Jose, CA) to >95% post sort purity. Cells were cultured at 10^5^ cells/well in a 96 well U bottom plate for 6 days in serum free X vivo medium (Lonza, Walkersville, MD) and stimulated with plate bound anti CD3(UCHT1; 5 ug/ml) and soluble anti CD28 (28.2; 1 ug/ml). In Th polarization assay, at the start of the culture, cells were differentiated with recombinant IL-2 (50 U/ml) for Th0, TGFβ1 (5 ng/ml), IL-1β (12.5 ng/ml), IL-6, IL-21 and IL-23 (all at 25 ng/ml) for Th17 in the presence and absence of potassium chloride.

### Flow-cytometry

For intracellular staining, cells cultured under Th17 condition on Day 7 were restimulated with PMA (phorbol 12-myristate 13-acetate; 50 ng/ml; Sigma-Aldrich), ionomycin (500 ng/ml; Sigma-Aldrich) and Golgi stop (BD Biosciences) for 4 h at 37 °C. Cells were stained with live-dead stain for 5 min at 4 °C to gate-out dead cells. The cells were then fixed and made permeable with Intracellular PermWash Buffer (Biolegend) for 15 min on ice followed by intracellular cytokine staining. Data were acquired on a FACSVerse (BD Biosciences) and analysed using FlowJo software (Tree Star, Inc., Ashland, OR).

#### Data record and statistical analysis

Continuous variables were presented as mean ± standard deviation (SD) or median (inter-quartile range) as appropriate. Categorical variables were compared using chi squared test. The continuous variables among controls and cases were compared using *unpaired t* test or Man Whitney U test depending upon normal or non-normal distribution. The comparison between same group of patients at different time points was done using paired t test or Wilcoxon rank-sum test. A P value of ≤0.05 was considered as statistically significant. Data analysis was done using SPSS version 24.

## Results

**Figure 1:**
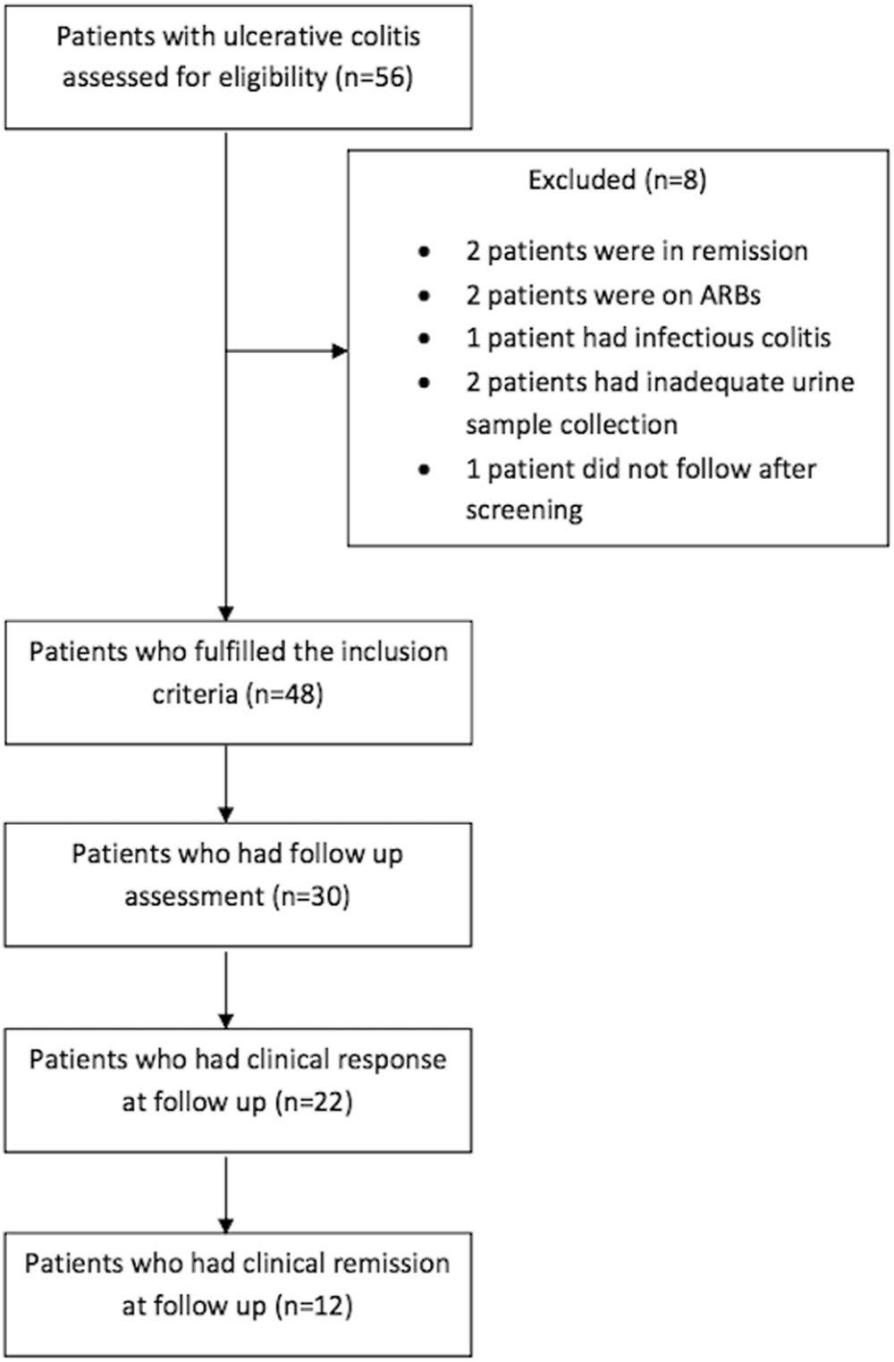
Study flowchart.

Fifty-six patients of ulcerative colitis were screened for inclusion and total of 48 patients fulfilled the inclusion criteria. Of these 30 patients had follow up assessment after a mean of 4.2 ± 2.7 months (Fig. 1).

### Baseline clinical and demographic characteristics

The median age of patients was 40 (IQR:28–46) years and that of controls was 43 (IQR: 35–47) years. Males were slightly more than females both among the patients (17:13) and controls (11:7). The median duration of disease was 3 (IQR: 2–10) years (Table 1). Four patients (13.3%) had E1 disease, 16 patients had E2 disease (53.3%) and 10 (33.3%) patients had E3 disease. Eight patients had mild (25%), and 22 (68.7%) had moderate to severe disease activity.

**Table 1:** Demographic and clinical profile of patients and their baseline biochemical parameters.

Parameter	Values
Age (in years)	40 (28–46)
Gender (M:F)	17:13
Disease Duration (years)	3 (2–10)
Hemoglobin (g%)	12.5 (10.9–13.2)
Platelets (x10^3^/cumm)	239 (200–330)
Albumin (g%)	4.3 (4–4.8)
Serum potassium (meq/L)	4.2 (3.9–4.6)
Disease extent	
• Proctitis	4 (13.3%)
• Left sided colitis	16 (53.3%)
• Pancolitis	10 (33.3%)
Disease severity	
• Mild	8 (26.8%)
• Moderate to severe	22 (73.2%)
Treatment given	
• 5-ASA	30 (100%)
• Steroids	6 (20%)
• Immunomodulators	5 (16.7%)
• Topical 5-ASA	13 (43.3%)
• Topical steroids	12 (40%)

All continuous variables are expressed as Median (Inter-quartile range)

5-ASA: 5 aminosalicylic acid.

### Comparison of fecal calprotectin and 24 hours urinary potassium levels among UC patients and controls

The level of intestinal inflammation was assessed by median fecal calprotectin level (370.28 ± 430.47 vs 12.44 ± 27.51, p < 0.001) which was found to be significantly higher in UC patients than controls (Table 2). Further a higher FCP value was accompanied by a significantly lower 24 hour urinary potassium levels in UC patients than controls (26.6 ± 16.9 vs 46.89 ± 35.91, p = 0.006).

**Table 2:** Fecal calprotectin, and 24 hours urinary potassium levels in UC patients and controls.

	UC (n = 30)	Controls (n = 18)	P Value*
Fecal Calprotectin (μg/g)	370.28 ± 430.47	12.44 ± 27.51	<0.001*
Urinary K (meq/L/24 hours)	26.6 ± 16.9	46.89 ± 35.91	0.01*

*P value significant at level of ≤0.05.

### Comparison of baseline and follow up fecal calprotectin, 24-hours urinary potassium, CRP and Mayo score in UC patients who had clinical response

**Figure 2:**
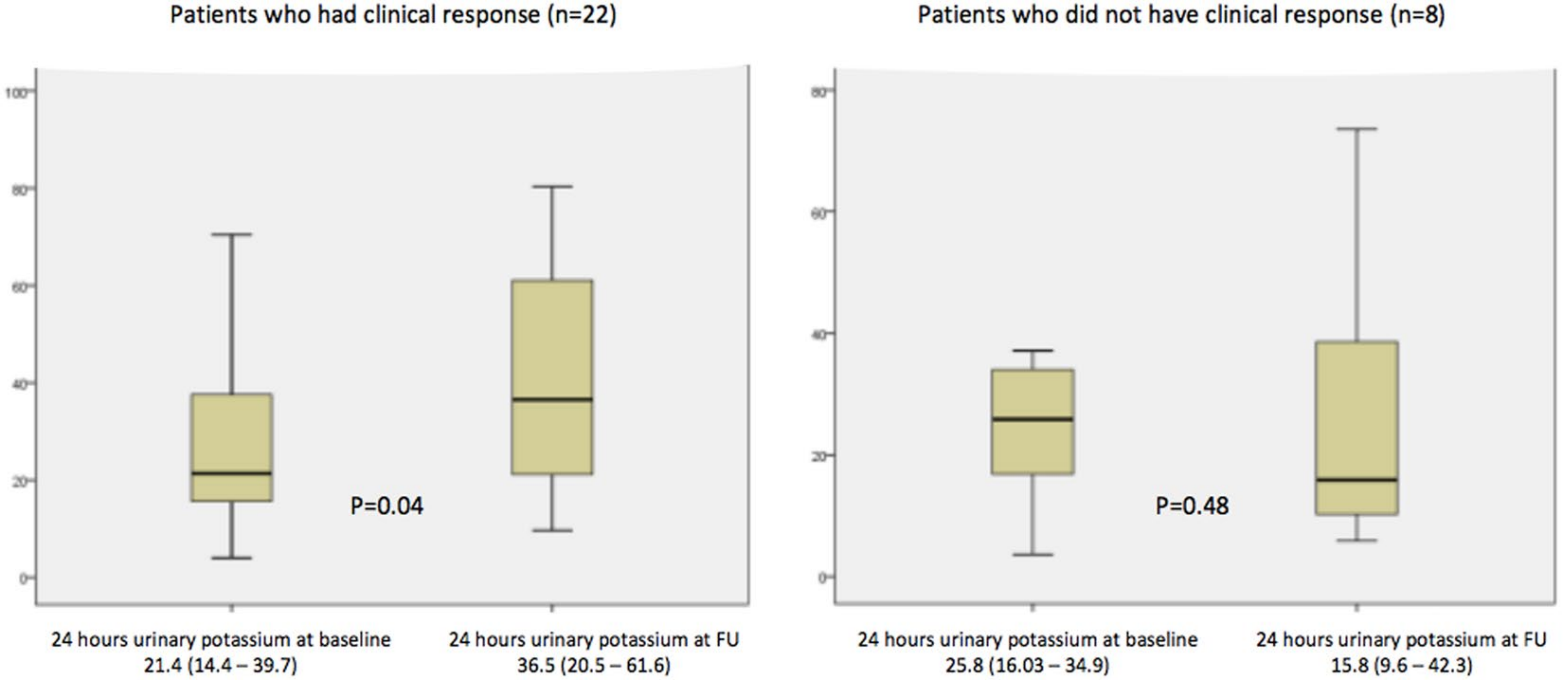
Box plot comparing median 24-hour urinary potassium levels at baseline and at follow-up in patients with and without clinical response.

**Figure 3:**
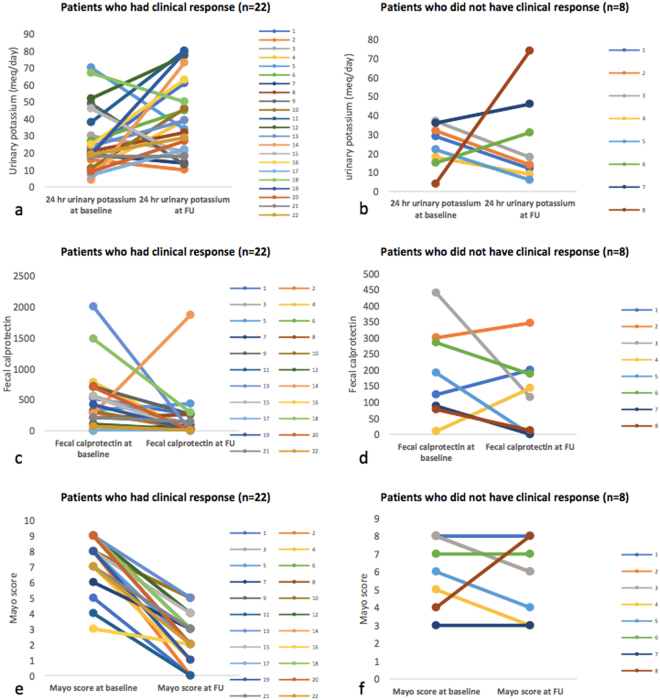
Per patient change in urinary potassium (**a**,**b**), fecal calprotectin (**c**,**d**) and Mayo score (**e**,**f**) among patients with clinical response vs. those without clinical response.

Of 30 patients, 22 had clinical response (Mayo score decline by $\ge$3). Among the patients who had clinical response, the median Mayo score [8 (7–9) to 2 (1.7–3.2), p < 0.001] and FCP levels decreased [302 (53–595) to 28 (5–261), p = 0.01] significantly at follow up, whereas no such trend was observed in patients who did not have clinical response (Table 3). 24-hour urinary potassium levels were significantly higher at follow up among patients with clinical response ([21.4 (14.2–39.7) to 36.5 (20.5–61.6), p = 0.04 (Fig. 2). There was no difference in the CRP levels at baseline and at follow up in both the groups. The per-patient change in Mayo score, FCP and urinary potassium in patients with clinical response vs those who did not have are depicted in Fig. 3.

**Table 3:** Comparison of median fecal calprotectin, 24 hours urinary potassium, CRP, Mayo score in 30 patients on presentation and follow up according to treatment response (responders vs non-responders.

Parameters	Patients who had clinical response (n = 22)			Patients who did not have clinical response (n = 8)		
	Baseline	Follow up	P value	Baseline	Follow up	P value*
Mayo score	8 (7–9)	2 (1.7–3.2)	<0.001	6.5 (4.2–8)	6 (3.2–7.8)	0.48
Urinary K (meq/L/24 hours)	21.4 (14.4–39.7)	36.5 (20.5–61.6)	0.04	25.8 (16.03–34.9)	15.8 (9.6–42.3)	0.48
Fecal Calprotectin (μg/g)	302 (53–595)	28 (5–261)	0.01	157 (39–296)	129 (3–196)	0.26
CRP (mg/L)	2.7 (1.2–6.2)	2.7 (0.8–6.6)	0.65	1.6 (1.4–13.7)	2.1 (0.5–3.5)	0.89

### Comparison of baseline and follow up fecal calprotectin, 24 hours urinary potassium, CRP and Mayo score in UC patients who had clinical remission

**Figure 4:**
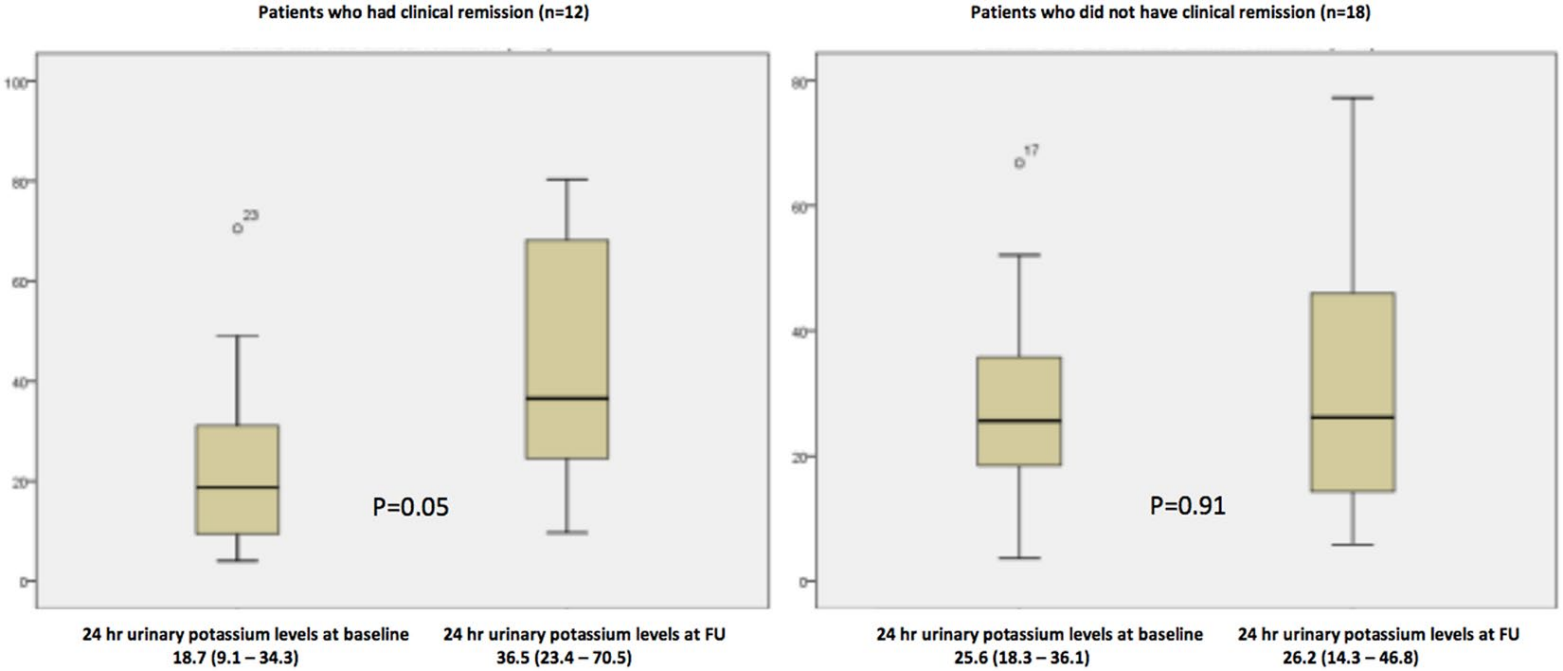
Box plot comparing median 24-hour urinary potassium levels at baseline and at follow-up in patients with and without clinical remission.

Of 30 patients, 12 achieved clinical remission (Mayo score <3). Patients with clinical remission at follow up had a significantly decreased median Mayo score [8 (5.5–9) to 2 (0.25–2), p < 0.01] along with a decreased FCP levels [312 (88–655) to 15.5 (1.8–256), p = 0.16], although the difference did not achieve statistical significance (Table 4). However, 24-hour urinary potassium levels were significantly higher at follow up among patients with clinical remission [18.7 (9.1–34.3) to 36.5 (23.4–70.5), p = 0.05] (Fig. 4).

**Table 4:** Comparison of fecal calprotectin, 24 hours urinary potassium, CRP, Mayo score in 30 patients on presentation and follow up according to treatment response.

Parameters	Patients who had clinical remission (n = 12)			Patients who did not have clinical remission (n = 18)		
	Baseline	Follow up	P value	Baseline	Follow up	P value*
Mayo score	8 (5.5–9)	2 (0.25–2)	0.002	8 (6–9)	4 (3–6)	0.004
Urinary K (meq/L/24 hours)	18.7 (9.1–34.3)	36.5 (23.4–70.5)	0.05	25.6 (18.3–36.1)	26.2 (14.3–46.8)	0.91
Fecal Calprotectin (μg/g)	312 (18–655)	15.5 (1.8–265)	0.169	210 (85–467)	90 (9–190)	0.016
CRP (mg/L)	4.7 (1.9–6.4)	1.5 (0.5–7.9)	0.33	1.6 (1.0–5.7)	2.5 (1.1–5.1)	0.74

### Comparison of baseline and follow up fecal calprotectin, 24-hours urinary potassium, CRP and Mayo score in UC patients who had mucosal healing vs those who did not have

Of 30 patients, 19 achieved mucosal healing (Mayo endoscopic sub-score ≤1). Patients with mucosal healing at follow up had a significantly decreased median Mayo score [8 (7–9) to 2 (1–3), p < 0.01] and decreased FCP levels [332 (106–700) to 30 (7–260), p = 0.02] (Table 5). However, 24 hour urinary potassium levels were increased at follow up among patients with mucosal healing [21.6 (10.7–46.1) to 38.8 (18.3–61.1), p = 0.12] although the difference did not achieve statistical significance.

**Table 5:** Comparison of fecal calprotectin, 24 hours urinary potassium, CRP, Mayo score in 30 patients on presentation and follow up according to presence or absence of mucosal healing.

Parameters	Patients who had mucosal healing (n = 19)			Patients who did not have mucosal healing (n = 11)		
	Baseline	Follow up	P value	Baseline	Follow up	P value*
Mayo score	8 (7–9)	2 (1–3)	<0.001	7 (4–8)	5 (3–7)	0.12
Urinary K (meq/L/24 hours)	21.6 (10.7–46.1)	38.8 (18.3–61.1)	0.12	22.4 (18.5–32.2)	29.3 (11.5–45.9)	0.86
Fecal Calprotectin (μg/g)	332 (106–700)	30 (7–260)	0.02	123 (70–285)	115 (0–200)	0.24
CRP (mg/L)	2.7 (1.3–6.0)	2.7 (0.7–6.3)	0.44	1.6 (0.7–17)	2.4 (0.6–4.4)	0.93

#### Correlation of change in Mayo score with change in fecal calprotectin levels and change in 24-hour urinary potassium levels

There was a statistically significant correlation between change in Mayo score and change in urinary potassium levels (r = 0.38, p = 0.04). However, there was no correlation between FCP change and urinary potassium change (r = −0.028, p = 0.89), as well as change in Mayo score and FCP change (−0.068, p = 0.73).

#### Potassium induces Foxp3^+^ CD4^+^ T cells under Th17 polarizing condition

**Figure 5:**
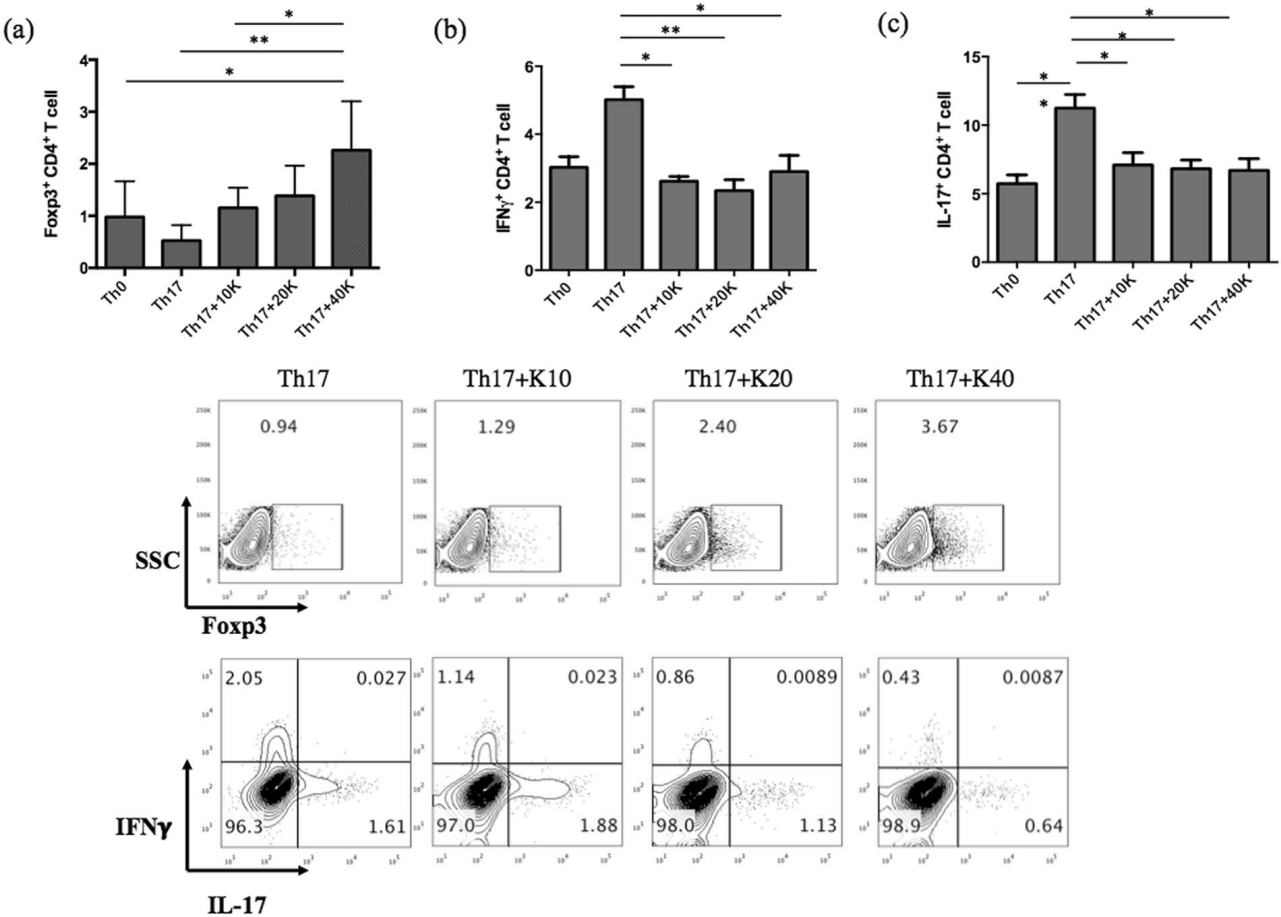
Sorted CD4^+^ T cells were stimulated for 6 days in the presence of anti CD3, anti CD28, hIL-2, Th17 polarizing condition in the presence of different doses of potassium and analysed for intracellular (**a**) Foxp3 (**b**) IFNγ and (**c**) IL-17. Percentages of IFNγ^+^, IL-17^+^ and Foxp3^+^ are represented as mean ± S.E.M of five independent experiments. *P < 0.05, **P < 0.001.

To investigate the role of potassium on inflammatory or tolerogenic responses of CD4^+^ T cells, we stimulated sorted CD4+ T cells with Th17 polarizing conditions in the presence of increasing doses of potassium (10, 20 and 40 nM). As hypothesized, potassium significantly reduced the IL-17 and IFNγ expression on Th17 cells (Fig. 5(b) and (c)). Surprisingly, it also promoted the Foxp3 expression (Fig. 5(a)) in a dose dependent manner. These data suggest that potassium suppresses the inflammatory response while promoting anti-inflammatory FOXP3^+^ CD4^+^ T cells under inflammatory environment (Th17).

## Discussion

The immunomodulatory action of the electrolytes- Na^+^ and K^+^ on inflammation has been studied in patients with cardiovascular risk^18^, cancer and chronic kidney disease patients and off late in IBD patients. Salt is an integral part of modern diet across the globe. However, in recent years, high salt intake has been associated with altered immune system leading to activated effector T cell responses. Both murine and human studies have depicted the role of NaCl in inducing Th17 related molecules while suppressing the Treg function via SGK1 signaling, thereby developing a more severe colitis in mice and aggravated disease activity in humans^19^. On the contrary, potassium enhanced the generation of FoxP3^+^ Treg cells in the presence of TGFβ1 and reinforced the Foxp3 expression in Th17 cells by activating Smad2/3 and inhibiting Smad 7 expression^20^. In mouse model of CKD, potassium supplementation down-regulates NF-κB and TGF β pathways and up-regulates Smad 7, thereby shifting the balance against pro-inflammatory state^21^. In another human report, potassium supplementation reduced IL-17A which was markedly enhanced by a high salt diet by inhibiting p38/MAPK-SGK1 pathway^22^. A recent study linked potassium with IBD and showed that dietary potassium, but not sodium, was inversely correlated with risk of Crohn’s disease in two large prospective cohorts of US women^20^. With a vast knowledge about the pro-inflammatory role of sodium, the role of potassium still has to be elucidated in IBD. With this background, we investigated the role of urinary potassium as a marker of intestinal inflammation in patients with ulcerative colitis. Also, as the previous two studies have highlighted the anti-inflammatory role of potassium, it would be very interesting to further take an intestinal inflammatory model such as ulcerative colitis patients and investigate the association of potassium with the disease severity in these patients.

The first major finding in our study was that the levels of 24-hour urinary potassium were significantly lower whereas FCP levels were significantly higher in patients with UC as compared to controls, thereby substantiating our hypothesis that levels of potassium had an inverse association with the disease activity in state of inflammation.

The second major finding was the significant increase of urinary potassium levels among UC patients who had a clinical response (decline in Mayo score ≥3) at follow up. This was accompanied by a significant parallel decline in Mayo score and fecal calprotectin levels. However, patients with no clinical response at follow up, had no change either in FCP levels or urinary potassium levels. These findings again strengthened the relationship of potassium to inflammation in IBD and revealed that the potassium levels increased with a decline in inflammation whereas they remained constant in patients who did not respond to therapy. Further studies are warranted to investigate whether there is a decline in potassium levels with concurrent relapse in the patient. This would further strengthen the hypothesis that urinary potassium estimation could be served as a marker of disease activity in IBD. Although the numbers are small, the results provide a signal which needs to be explored further. Similar trends were observed among patients who had clinical remission and mucosal healing.

We then corroborated our findings by studying the *in vitro effect* of potassium on inflammatory responses of CD4^+^ T cells. We observed that even in the face of Th17 polarizing conditions potassium significantly increased the expression of FoxP3 from Th17 cells in a dose dependent manner which was similar to a previous study by Khalili *et al*.^20^. However, our main focus was to check the effect of potassium on the major pro-inflammatory cytokines which are involved in aggravating the intestinal inflammation. We found that potassium reduced the expression of IL-17 and interferon $\gamma$ which strenghthened our findings that potassium established a tolerogenic response while suppressing the inflammation inducing cytokines.

This is the first proof of concept study that documents an association between disease activity in UC and urinary potassium levels. However, there are several limitations associated with this study. The patient numbers were small, but we had the follow-up results for all the patients, and even with this small number we had significant results. We did not control the dietary pattern but measured the urinary potassium which indirectly reflects the amount of the electrolyte present in the body. This potential confounder can be offset by the fact that the patients served as their own control and measuring potassium levels in the same patient at different time points (baseline and follow up) would also negate the minimal effect of diet on urinary potassium levels. Also, diet has a major influence on the urinary sodium levels, whereas potassium levels remain largely unaffected by dietary perturbations^23^. There was also an evident difference in the changes in urinary potassium between responders and non-responders, and the change in urinary potassium correlated with change in Mayo score (r = 0.38, p = 0.04). 24-hour urine collection has its own fallacies in being cumbersome for patients and there could be chances of error in collection. Therefore, estimating changes in spot urine samples could be a better approach in this direction^24^. We also did not assess the post-therapy serum albumin and 24-hour urinary excretion of albumin in our patients, as assessment of nutritional and volume status after therapy (i.e. post-therapy albumin and total 24 hour urinary excretion) would vary between responders and non-responders.

In conclusion, we have shown that urinary potassium level is inversely associated with the disease activity in ulcerative colitis and *in vitro* data suggests that it suppresses inflammation by reducing the IL-17 and IFNγ and inducing the Foxp3 expression on Th17 cells. This study has laid a platform for further research in this field exploring the potential of urinary potassium as a biomarker for intestinal inflammation.
